# Neural Systems Underlying Emotional and Non-emotional Interference Processing: An ALE Meta-Analysis of Functional Neuroimaging Studies

**DOI:** 10.3389/fnbeh.2016.00220

**Published:** 2016-11-15

**Authors:** Min Xu, Guiping Xu, Yang Yang

**Affiliations:** ^1^Neuroimaging Laboratory, School of Biomedical Engineering, Shenzhen University Health Science CenterShenzhen, China; ^2^Center for Neuroimaging, Shenzhen Institute of NeuroscienceShenzhen, China; ^3^Guangdong Key Laboratory of Biomedical Information Detection and Ultrasound Imaging, Shenzhen UniversityShenzhen, China; ^4^Department of Psychology, Guangdong University of EducationGuangzhou, China; ^5^Department of Linguistics, School of Humanities, The University of Hong KongHong Kong, China

**Keywords:** interference processing, meta-analysis, emotional interference, non-emotional interference, activation likelihood estimation, fMRI

## Abstract

Understanding how the nature of interference might influence the recruitments of the neural systems is considered as the key to understanding cognitive control. Although, interference processing in the emotional domain has recently attracted great interest, the question of whether there are separable neural patterns for emotional and non-emotional interference processing remains open. Here, we performed an activation likelihood estimation meta-analysis of 78 neuroimaging experiments, and examined common and distinct neural systems for emotional and non-emotional interference processing. We examined brain activation in three domains of interference processing: emotional verbal interference in the face-word conflict task, non-emotional verbal interference in the color-word Stroop task, and non-emotional spatial interference in the Simon, SRC and Flanker tasks. Our results show that the dorsal anterior cingulate cortex (ACC) was recruited for both emotional and non-emotional interference. In addition, the right anterior insula, presupplementary motor area (pre-SMA), and right inferior frontal gyrus (IFG) were activated by interference processing across both emotional and non-emotional domains. In light of these results, we propose that the anterior insular cortex may serve to integrate information from different dimensions and work together with the dorsal ACC to detect and monitor conflicts, whereas pre-SMA and right IFG may be recruited to inhibit inappropriate responses. In contrast, the dorsolateral prefrontal cortex (DLPFC) and posterior parietal cortex (PPC) showed different degrees of activation and distinct lateralization patterns for different processing domains, which suggests that these regions may implement cognitive control based on the specific task requirements.

## Introduction

In our daily life, we consistently need to adapt our behavior by focusing cognitive resources on goal-relevant information while filtering out irrelevant information that can interfere with the appropriate response (Desimone and Duncan, 1995; Botvinick et al., 2001; Ridderinkhof et al., 2004; Nee et al., 2007). Such capacity is essential for human adaptation (Mansouri et al., 2009). In the neuroimaging literature, interference control has often been linked to neural activations in the anterior cingulate cortex (ACC), dorsolateral prefrontal cortex (DLPFC), posterior parietal cortex (PPC), and anterior insula. The ACC is proposed to monitor the conflict (Botvinick et al., 2001; Carter and Van Veen, 2007), or adjust the level of cognitive control to resolve interference (Posner and DiGirolamo, 1998; Roelofs et al., 2006). The DLPFC is proposed to contribute to top-down control, leading to a bias in processing task-relevant information when there is competition between two representations of stimuli (MacDonald et al., 2000; Egner and Hirsch, 2005; Mansouri et al., 2009). The PPC has been suggested to subserve top-down regulation of attention (Roberts and Hall, 2008), action planning when there is conflict response (Coulthard et al., 2008), and cognitive control at the level of stimuli representation (Liston et al., 2006). The role of the anterior insula in interference processing is less clear, yet it has been shown to be associated with response selection (Wager et al., 2005), inhibiting inappropriate responses (Garavan et al., 1999), monitoring (Cieslik et al., 2015), error awareness (Ullsperger et al., 2010), and goal-directed attention (Craig, 2009; Tops and Boksem, 2011).

A wide range of tasks have been used to investigate interference processing, such as the Stroop task (Stroop, 1935; MacLeod, 1991), the Simon task (Simon and Berbaum, 1990), the stimulus-response compatibility (SRC) task (Fitts and Seeger, 1953), Eriksen Flanker task (Eriksen and Eriksen, 1974), and so forth. To perform these tasks, participants have to make a context-appropriate response while inhibiting the tendencies to process irrelevant information and to make a competing automatic response. Understanding whether and how the nature of interference might influence the recruitment of neural systems is considered as the key to understanding the brain organization of cognitive control (Barch et al., 2001; Fan et al., 2003; Wittfoth et al., 2006, 2009; Nee et al., 2007; Roberts and Hall, 2008; Hedden and Gabrieli, 2010; Jiang and Egner, 2014). In an fMRI study, Barch et al. found that the same regions of ACC were responsive to interference induced by different response modalities (i.e., vocal vs. manual) and to interference in different processing domains (i.e., verbal vs. spatial) (Barch et al., 2001). Another fMRI study compared brain activation for the color-word Stroop task (which addresses verbal interference processing), and Simon and Flanker tasks (which tap spatial interference) (Fan et al., 2003). Results showed that both common (ACC and prefrontal cortex) and distinct brain systems supported different tasks. Thus, it is plausible to suggest that there might be a single network for conflict monitoring but different sites for conflict resolution according to the specific requirements of the tasks.

Although functional neuroimaging studies have provided ample information about the brain systems for interference processing, there are limitations, such as small sample size and low reliability (Raemaekers et al., 2007; Wager et al., 2007; Eickhoff et al., 2012). Consequently, several meta-analyses have been performed on neuroimaging studies that used different experimental paradigms to identify neural systems underlying interference processing (Derrfuss et al., 2005), as well as common and distinct neural systems associated with distinguishable stages or sub-components of interference processing (Nee et al., 2007; Niendam et al., 2012; Cieslik et al., 2015). For instance, Derrfuss et al. (2005) performed meta-analyses with 14 fMRI studies using task-switching paradigm, and with 11 studies using the color-word Stroop task. Results showed that the left inferior frontal junction was consistently activated in task-switching and Stroop paradigms, suggesting that these regions played an important role in updating task representations. Nee et al. (2007) conducted a peak density-based meta-analysis on 47 neuroimaging studies involving a wide range of interference tasks, including the color-word Stroop, Flanker, Simon, SRC, go/no-go, and stop signal tasks. The authors identified the ACC, DLPFC, inferior frontal gyrus (IFG), PPC and anterior insula to be important for interference resolution, and proposed that separate interference resolution mechanisms acted upon different stages of processing, with the right IFG heavily involved in response execution, the right DLPFC and ACC in response selection and the left DLPFC in resolution of stimulus conflict. A recent meta-analysis study by Cieslik et al. (2015) compared convergence of activation for four subcategories of cognitive control, i.e., go/no-go tasks assessing action withholding, stop-signal tasks assessing action cancelation, Stroop, and spatial interference tasks assessing interference control. They found high convergence of activation across paradigms in the right anterior insula, inferior frontal junction and anterior midcingulate cortex, suggesting that these regions might play a regulatory role in a process-general manner.

Recent years have seen a surge of interest in investigating emotional interference processing, and neuroimaging studies have reported altered emotional and non-emotional interference processing in patients with major depression disorders (Fales et al., 2008; Etkin and Schatzberg, 2011; Chechko et al., 2013), posttraumatic stress disorder (Bremner et al., 2004; Shin et al., 2007), bipolar disorder (Kronhaus et al., 2006; Rey et al., 2014) and so forth. Comparing neural systems engaged in emotional vs. non-emotional interference processing would help differentiate brain areas associated with domain-general control processes from those that are associated with a specific domain, which would aid the interpretation of results from patient studies. Neuroimaging evidence has suggested that both emotional and non-emotional conflict recruit the dorsal ACC in conflict detection, but regarding conflict resolution, the ventral ACC is involved in resolving emotional conflict whereas the lateral prefrontal cortex is involved in resolving cognitive conflict (Egner et al., 2008).

To our knowledge, there are no meta-analyses examining whether the same neural networks are recruited in emotional and non-emotional interference processing. Although, there are previous meta-analysis studies describing neural systems for both cognitive and emotional domains (Shackman et al., 2011; Diener et al., 2012; Cromheeke and Mueller, 2014), they have included studies that were not associated with emotional interference processing *per se*. For example, in the meta-analysis by Shackman et al. (2011), only studies with manipulations designed to induce negative emotions were included, while those studies involving interference processing in the emotional domain were excluded to ensure a clear-cut effect between cognition and emotion.

In our study, we carried out a quantitative meta-analysis using the activation likelihood estimation (ALE) method for neuroimaging studies (Turkeltaub et al., 2002; Laird et al., 2005b) to assess significant convergent and divergent patterns of brain activity associated with emotional and non-emotional interference processing. Neuroimaging studies using the face-word conflict task were included to examine neural co-activation patterns associated with emotional interference. Moreover, to get a more comprehensive picture of the domain-general and domain-specific neural systems, we included two subcategories of non-emotional interference, i.e., non-emotional verbal interference with the color-word Stroop task and non-emotional spatial interference with the Simon, SRC, and Flanker tasks.

## Methods

### Paradigms included

#### Face-word conflict task

In the domain of emotional interference, we included a face-word (or picture-word) conflict task, in which the emotional conflict is manipulated by varying the congruency between an emotional expression and a word written on the picture. For example, subjects may be asked to make response to “fear” or “happy” for the affect expressed on the face. A conflict arises if the word “fear” is shown together with a happy face or the word “happy” across a face displaying fear. The face-word conflict task provides a measure of emotional conflict comparable to that of the color-word Stroop task (Etkin et al., 2006). It should be noted that, while there are studies using an emotional Stroop task, in which subjects are required to identify the ink color of words that are either emotional neutral or emotional negative (McKenna, 1986; Williams et al., 1996), this task does not involve an effect of conflict between task relevant (i.e., word color) and task-irrelevant emotional dimension of the stimuli (Algom et al., 2004). Consequently, we did not include this paradigm in our meta-analysis.

#### Color-word stroop task

The color-word Stroop task induces a conflict between stimulus color and word meaning (Stroop, 1935; MacLeod, 1991). In a typical task, subjects are required to identify the ink color of a word, whereas the word itself is the name of a color. Interference arises when the ink color and the word meaning are incongruent (e.g., the word RED printed in green ink), resulting in response with longer reaction times and higher error rates than for the congruent or neutral condition.

#### Simon task

Conflict in the Simon task arises from the mismatch between stimuli location and the direction of response. For example, subjects may be asked to make a right-hand keypress to a red circle and left-hand keypress to a green circle while ignoring the location of the stimulus. It is found that reaction times are longer when the red circle appears on the left side or the green circle on the right side of the screen, due to the interference caused by the spatial dimension of the stimulus.

#### SRC task

Unlike the Simon task, in which stimuli location is task-irrelevant, the SRC task has stimulus spatial features assigned to their corresponding response (congruent mapping) or to the opposite response (incongruent mapping). For instance, in the congruent condition subjects may be instructed to make a left-hand keypress to an arrow pointing left and a right-hand keypress to an arrow pointing right, whereas in the incongruent condition they need to make a left-hand keypress to an arrow pointing right and a right-hand keypress to an arrow pointing left. The subjects are found to perform more quickly and more accurately when the mappings of stimuli and responses are congruent than when they are incongruent (Proctor and Vu, 2006)

#### Flanker task

In the Flanker task, subjects are required to respond to a central stimulus while ignoring flanking stimuli. The conflict is induced when the flanking stimuli are associated with different responses from the central stimuli. For example, subjects may be asked to indicate the orientation of a central arrow, which is flanked by distractor arrows. In the congruent condition the flanking arrows pointed in the same direction (e.g., < < < < <) and in the incongruent condition in the opposite direction (< < > < <) as the central arrow. It has been shown that subjects involuntarily process the flanking stimuli despite their irrelevance to the task requirements (Botvinick et al., 1999).

### Article search and selection

Relevant articles published from 1 January 1994 through 1 January 2015 were identified through searching the PubMed database (http://www.pubmed.gov). Studies were included if they met all the following four criteria: (1) used fMRI; (2) reported whole-brain coordinates in MNI or Talairach space; (3) reported coordinates for the contrast incongruent condition > congruent condition or incongruent condition > neutral condition; (4) recruited healthy adults as subjects (for clinical studies, the data of the healthy control group was included if coordinates were reported for the healthy group). In addition, for the non-emotional spatial domain (i.e., Flanker, Simon task, or SRC tasks), studies using words or letters as stimuli were excluded. A total of 78 studies met our criteria. Table 1 lists the studies included in the meta-analysis. The selected studies were categorized according to experiment paradigms used. Specifically, the emotional verbal domain comprised studies using the face/picture-word conflict tasks (14 experiments from 13 articles, foci = 127, subjects = 285), non-emotional verbal domain included studies using the color-word Stroop task (35 experiments from 33 articles, foci = 374, subjects = 607), and non-emotional spatial domain included studies using the Simon, SRC or Flanker tasks (29 experiment from 24 articles, foci = 313, subjects = 527). The coordinates from the contrast incongruent > congruent were used (if the study did not report such contrast, the results of incongruent > neutral were used).

**Table 1:** **Studies included in the meta-analysis on interference processing**.

**1st author**	**Year**	**Control condition**	**N**	**Age**	**Task**
**EMOTIONAL VERBAL**					
Haas	2006	Congruent	13	21 (2.2)	Face-word
Park	2008	Congruent	14	25.9 (1.6)	Picture-word
FruÌĹhholz	2009	Congruent	20	22.7 (2.25)	Face-word
Chechko	2009	Congruent	18	30.0 (6.4)	Face-word
Ovaysikia	2011	Congruent	10	21–40	Face-word
	2011	Congruent	10	21–40	Face-word
Krug	2011	Congruent	30	25.4 (4.7)	Face-word
Krug	2012	Congruent	42	23.2 (4.6)	Face-word
Chechko	2012	Congruent	24	27.6 (4.1)	Face-word
Jarcho	2013	Congruent	35	19.68 (1.68)	Face-word
Torres-Quesada	2013	Congruent	21	24.8 (19–34)	Face-word
Chechko	2013	Congruent	18	36.0 (10.3)	Face-word
Offringa	2013	Congruent	18	27.06 (6.0)	Face-word
Rey	2014	Congruent	12	41.3 (12)	Face-word
**NON-EMOTIONAL VERBAL**					
Milham	2001	Neutral	16	18–30	Color-word
Zysset	2001	Congruent	9	21–34	Color-word
Ruff	2001	Neutral (color naming)	12	23.08 (2.48)	Color-word
	2001	Neutral (word naming)	12	23.08 (2.48)	Color-word
Steel	2001	Nonlexical	7	22–30	Color-word
Banich	2001	Neutral	14	21–35	Color-word
Milham	2002	Congruent, neutral	12	21–27	Color-word
Mead	2002	Congruent	18	26.7 (18–46)	Color-word
Adleman	2002	Nonlexical	11	20 (17.4–22.7)	Color-word
Norris	2002	Neutral	4	/	Color-word
Milham	2003	Neutral	16	/	Color-word
Fan	2003	Congruent	12	24.7 (4.6)	Color-word
Potenza	2003	Congruent	11	29 (7.81)	Color-word
Compton	2003	Neutral	12	25.2 (20–31)	Color-word
Kerns	2005	Congruent	13	36 (4.6)	Color-word
Mitchell	2005	Neutral	15	23.3 (6.31)	Color-word
Milham	2005	Congruent	18	18–40	Color-word
Van Veen	2005	Congruent	14	21.4 (2.2)	Color-word
Brass	2005	Congruent	10	21–37	Color-word
Coderre	2008	Congruent (Kana)	9	36 (9.4)	Color-word
	2008	Congruent (Kanji)	9	36 (9.4)	Color-word
Roberts	2008	Neutral	16	24.3 (16–42)	Color-word
Becker	2008	Congruent	17	32.7 (7.8)	Color-word
Mathis	2009	Neutral	12	26.8 (22–30)	Color-word
Prakash	2009	Neutral	25	23.6 (18–35)	Color-word
Zoccatelli	2010	Congruent	10	28 (22–40)	Color-word
Fechir	2010	Congruent	16	23.8 (1.4)	Color-word
Silton	2010	Congruent	30	18–34	Color-word
Polosan	2011	Congruent	14	35.9 (7.2)	Color-word
Grandjean	2012	Neutral	25	21.8 (2.68)	Color-word
Terry	2012	Congruent	20	20.4 (1.6)	Color-word
Grandjean	2013	Congruent	25	21.8 (2.68)	Color-word
Piai	2013	Congruent	23	18–29	Color-word
Ent	2014	Congruent	46	36.9 (8.9)	Color-word
Veroude	2013	Neutral	74	18–26	Color-word
**NON-EMOTIONAL SPATIAL**					
Hazeltine	2000	Congruent	8	21.0 (18–24)	Flanker
Maclin	2001	Congruent	8	18–47	Simon
Ullsperger	2001	Congruent	12	24.9 (21–29)	Flanker
Bunge	2002	Neutral	16	24 (19–33)	Flanker
Fan	2003	Congruent	12	24.7 (4.6)	Simon
	2003	Congruent	12	24.7 (4.6)	Flanker
Sylvester	2003	Congruent (event-related)	14	18–25	SRC
	2003	Congruent (blocked)	14	/	SRC
Liu	2004	Congruent	11	24–40	Simon
Wager	2005	Congruent	14	18–25	Flanker
	2005	Congruent	14	18–25	SRC
Kerns	2006	Congruent	26	24.2 (4.5)	Simon
Wittfoth	2006	Congruent (motion)	20	25.5 (21–31)	Simon
	2006	Congruent (location)	20	25.5 (21–31)	Simon
Schmitz	2006	Congruent	12	39 (6)	Simon
Rubia	2006	Congruent	21	28 (6)	Simon
Blasi	2006	Congruent, neutral	57	28.6 (6.0)	Flanker
Lungu	2007	Congruent	8	22 (20–38)	SRC
Fan	2007	Congruent	20	26 (18–59)	Flanker
Wittfoth	2008	Congruent	20	25.5 (21–31)	Simon
Forstmann	2008	Neutral	24	24.2 (2.76)	Simon
Fan	2008	Congruent	16	27.2 (5.7)	Flanker
Wittfoth	2009	Congruent	14	25 (2.6)	Simon
Zhu	2010	Congruent	22	20 (3)	Flanker
FruÌĹhholz	2011	Congruent	24	23.9 (5.3)	Flanker
	2011	Congruent	24	23.9 (5.3)	Simon
Sebastian	2012	Congruent	24	30.3 (8.1)	Simon
Korsch	2014	Congruent	20	22.95 (2.72)	Flanker
Korsch	2014	Congruent	20	22.95 (2.72)	SRC

N, number of subjects; SRC, stimulus-response compatibility.

### ALE analysis

ALE is used for coordinate-based meta-analyses of neuroimaging data and exploring brain systems that were most consistently activated across studies using similar tasks (Turkeltaub et al., 2002; Laird et al., 2005b). Activation foci reported in published studies are treated as 3D Gaussian probability distributions centered at the given coordinates. ALE maps are then constructed by computing the union of activation probabilities for each voxel. To determine the reliability of ALE maps, a permutation procedure is applied to test the differentiation between true convergence of foci and random clustering (Eickhoff et al., 2009, 2012; Turkeltaub et al., 2012). Here, ALE meta-analysis was conducted using GingerALE 2.3 (http://www.brainmap.org/). We first converted Talairach peaks into MNI space using the Lancaster transform as implemented in the GingerALE software package (Lancaster et al., 2007; Laird et al., 2010). A random effect, Turkeltaub Non-Additive method was used, which minimizes both within-experiment and within-group effects (Turkeltaub et al., 2012). Threshold was set at *p* <0.05 False Discovery Rate pN, with a minimum cluster size of 250 mm^3^ (Laird et al., 2005a). Three ALE maps were generated for the three processing domains, i.e., emotional verbal, non-emotional verbal, and non-emotional spatial interference. The overlap among the three processing domains was analyzed by performing pairwise conjunction analyses and a conjunction analysis across the three domains, using the voxel-wise minimum values of the thresholded ALE maps (Nichols et al., 2005). The differences in activation in the three processing domains were identified by pairwise subtraction analyses (Eickhoff et al., 2011). It should be noted that study sizes of the ALE datasets are corrected in the subtraction analyses. Specifically, all experiments in the contrast analysis were pooled and randomly divided into two groups of the same size as the original data sets. ALE images were created for the new data sets and the differences between them were computed and then compared to the true data. After 10,000 permutations, the analysis yields a null-distribution for the difference in ALE values and can see where the actual observed data's values sit on the distribution. In this study, all ALE coordinates were reported in MNI space. All images were overlaid onto standard brain in MNI space using Mango software.

## Results

### Individual ALE maps for the three domains of interference processing

**Figure 1:**
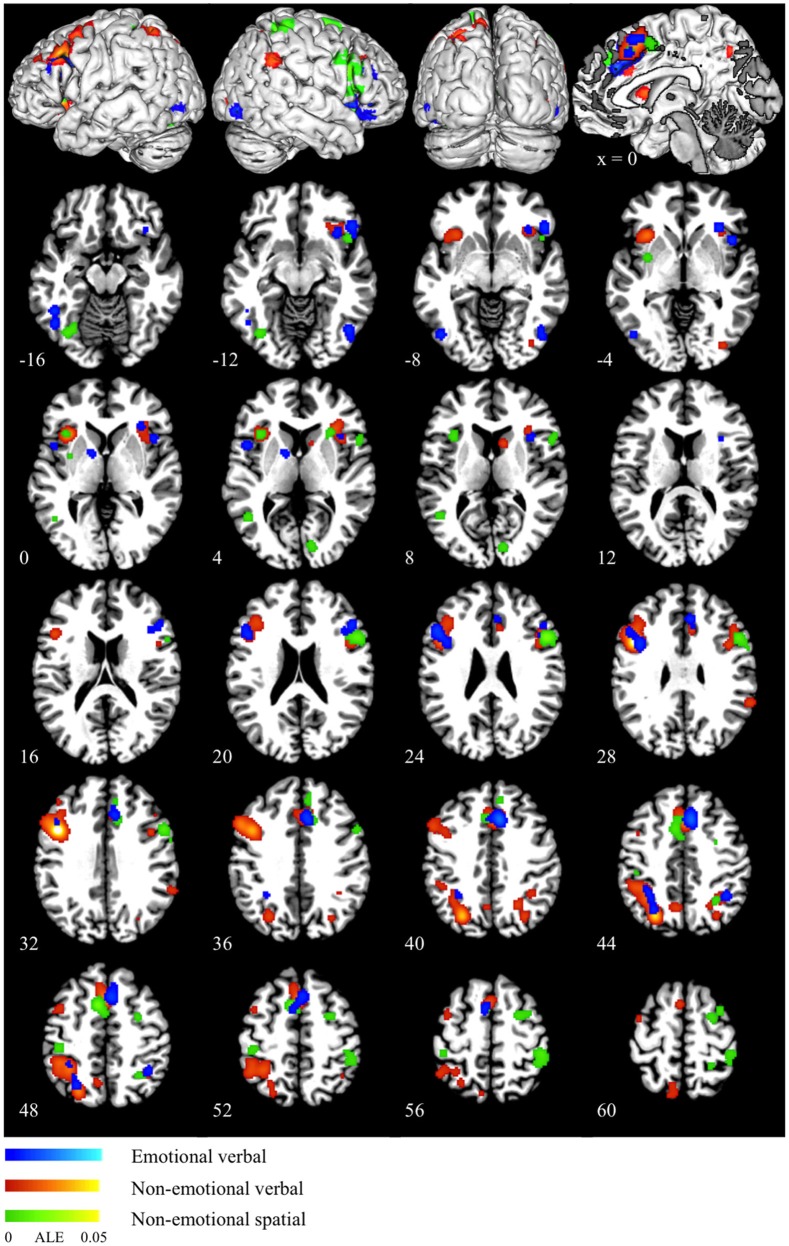
**ALE maps for emotional verbal (blue), non-emotional verbal (red) and non-emotional spatial (green) interference processing**. The scale color bar shows the ALE values in the range from 0 to 0.05 for all the ALE maps.

Meta-analysis of neuroimaging studies on the emotional interference processing showed the activation converged in the dorsal ACC at BA 32, presupplementary motor area (pre-SMA) at BA 6, bilateral insula (BA 13), bilateral inferior and middle frontal gyrus (BA 47/46/9), bilateral PPC (BA 40/7), bilateral fusiform gyrus (BA 19/37), right cerebellum and left lentiform nucleus (as shown in blue in Figure 1 and Table 2).

**Table 2:** **Peaks of convergence for the three domains of interference processing**.

**Regions**	**BA**	**Volume (mm^3^)**	**Coordinates**		
			**X**	**Y**	**Z**
**EMOTIONAL VERBAL**					
Cingulate gyrus/pre-SMA	32/6	4672	8	20	42
			6	32	28
			−6	6	54
L inferior/middle frontal gyrus	46/9	2144	−50	22	24
			−46	18	32
L posetrior parietal cortex	7/40	1344	−28	−64	46
			−32	−48	44
R inferior frontal gyrus	45	952	48	26	20
R insula	13	880	34	26	−4
R inferior frontal gyrus	47	864	50	28	−10
L fusiform gyrus	37	808	−48	−50	−16
R fusiform gyrus	19	752	46	−68	−10
R inferior parietal lobule	40	520	40	−50	46
R cerebellum	–	472	36	−50	−22
R insula	13	456	44	16	−2
L inferior temporal gyrus	19	416	−46	−72	−6
L insula	13	360	−48	8	2
L lentiform nucleus	–	304	−12	2	2
R insula	13	296	36	16	8
**NON-EMOTIONAL VERBAL**					
L inferior/middle frontal gyrus/precentral gyrus	9/46/6	10376	−44	10	30
			−40	28	22
			−42	8	48
			−40	−2	58
			−44	36	32
L precuneus/inferior/superior pareital lobule	19/40/7	8944	−26	−70	42
			−34	−48	48
			−28	−62	56
Pre-SMA/cingulate gyrus	6/32	6792	0	14	50
			4	20	40
			8	22	28
R insula/inferior frontal gyrus	13/47	2704	32	24	4
			36	24	−10
L insula	13	2656	−34	20	0
R inferior/middle frontal gyrus	45/44/9	2352	48	16	24
			42	8	30
R precuneus	7/19	2056	24	−58	40
			30	−70	40
L precuneus	7	560	−10	−72	58
L precuneus	7	536	−6	−62	44
R supramarginal gyrus	40	448	62	−46	30
R caudate	–	280	12	10	8
R inferior occipital gyrus	18	272	36	−82	−4
**NON-EMOTIONAL SPATIAL**					
Pre-SMA/cingulate gyrus	6/32	3328	−6	10	50
			12	20	34
			−6	20	42
R inferior/middle frontal gyrus/precentral gyrus	45/9/6	2632	52	16	24
			54	10	32
			60	2	30
R middle frontal gyrus/precental gyrus	6	1568	28	−4	64
			26	0	56
R inferior parietal lobule	40	1528	46	−34	56
L fusiform gyrus	19	984	−36	−70	−14
L inferior parietal lobule	40	720	−44	−30	48
L insula	13	648	−36	20	4
L middle temporal gyrus	37	464	−46	−58	4
R cuneus	17	464	12	−84	6
R inferior frontal/precentral gyrus	44	432	56	14	6
Medial frontal gyrus	8	416	8	38	38
R superior parietal lobule	7	376	26	−44	64
R inferior frontal gyrus	47	328	46	16	−12
R superior parietal lobule	7	320	30	−54	46
R insula	13	272	30	22	4
L superior parietal lobule	7	272	−14	−56	68
L putamen	–	256	−32	−2	−2

L, left; R, right; BA, Brodmann's area.

Non-emotional verbal processing using the color-word Stroop task was associated with high convergence of activation in the dorsal ACC (BA 32), pre-SMA (BA 6), bilateral anterior insula (BA 13), bilateral ventral IFG (AB 47), left DLPFC (BA 46/9), right inferior/middle frontal gyrus (BA 45/44/9), left precentral gyrus (BA 6), bilateral PPC (BA 7/40), right caudate and right inferior occipital gyrus (BA 18) (as shown in red in Figure 1 and Table 2). Activation in the DLPFC and superior parietal lobule was strongly left lateralized.

ALE maps for non-emotional spatial interference processing showed high convergence of activation mainly in the dorsal ACC (BA 32), pre-SMA (BA 6), bilateral insula (BA 13), right inferior, and middle frontal gyrus (BA 45/9), right precentral gyrus (BA 6), bilateral PPC (BA 40/7), left middle temporal gyrus (BA 37), left putamen, and left cerebellum (as shown in green in Figure 1 and Table 2). In contrast to the left-lateralized activity for the non-emotional verbal interference processing, the activity associated with non-emotional spatial interference was predominantly right lateralized, particularly in the prefrontal cortex.

### Conjunction and subtraction analyses

**Figure 2:**
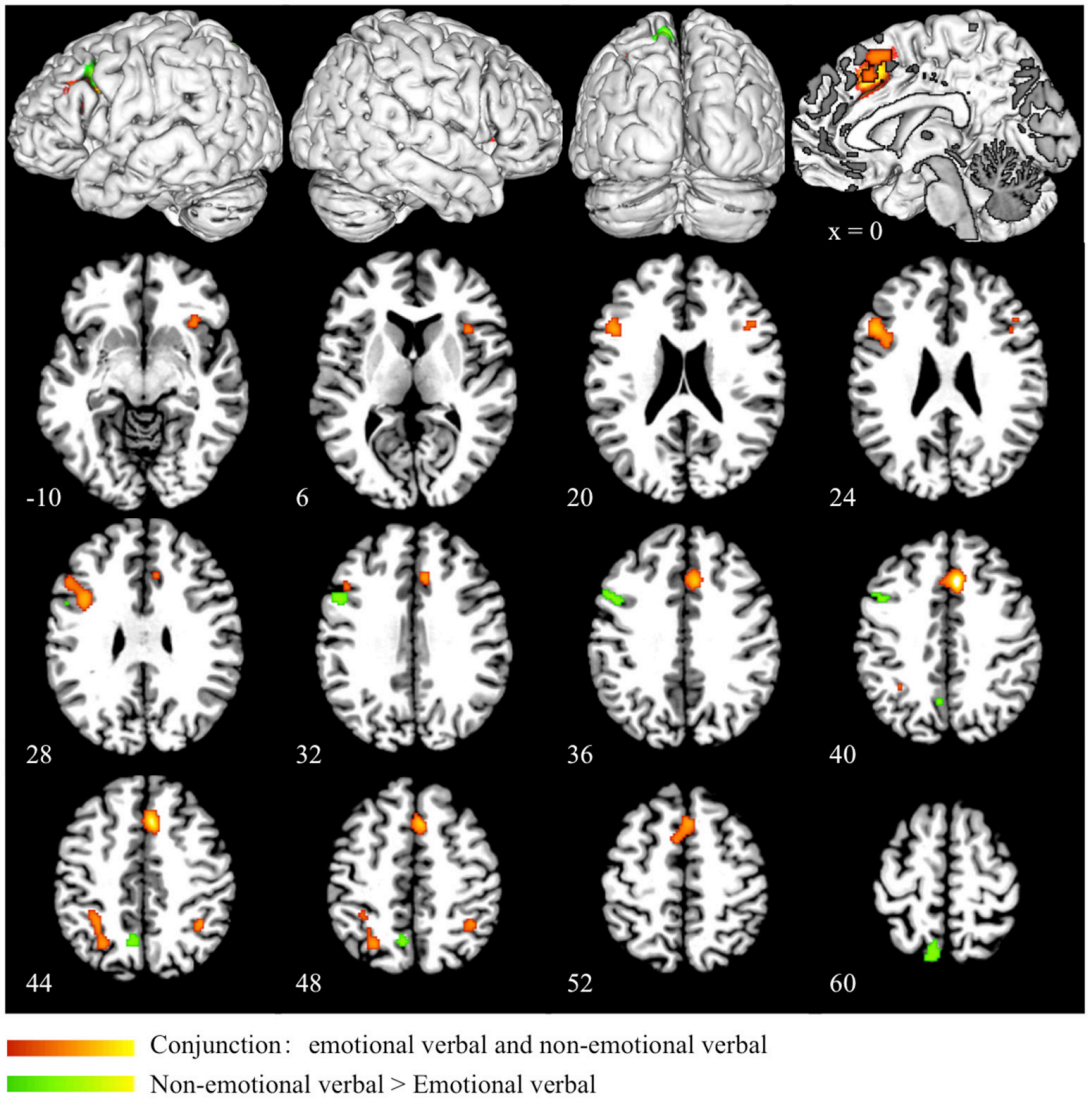
**Conjunction and subtraction analyses of emotional verbal and non-emotional verbal interference processing**. The scale bar in red represents minimum ALE values from 0 to 0.022 in the conjunction analysis. The scale bar in green represents *z*-values from 0 to 6 for the contrast of non-emotional verbal > emotional verbal domain. No significant cluster is found for the contrast of emotional verbal > non-emotional verbal.

Conjunction analysis of emotional verbal and non-emotional verbal interference processing showed common activation mainly in the dorsal ACC and pre-SMA (BA 32/6), right insula (BA 13), right IFG (BA 47), left DLPFC (BA 46/9), left precuneus (BA 7), and bilateral inferior parietal lobules (BA 40) (as shown in red in Figure 2 and Table 3). A direct contrast between the two domains showed greater activation for the non-emotional verbal processing in the left inferior/middle frontal gyrus (BA 9) and left precuneus (BA 7) (as shown in green Figure 2 and Table 3). No increased activation was found for emotional interference processing.

**Table 3:** **Peaks of convergence for conjunction and subtraction analyses**.

**Regions**	**BA**	**Volume (mm^3^)**	**Coordinates**		
			**X**	**Y**	**Z**
**CONJUNCTION: EMOTIONAL VERBAL AND NON-EMOTIONAL VERBAL**					
Cingulate gyrus/pre-SMA	32/6	3064	6	20	42
			−6	8	54
L inferior/middle frontal gyrus	46/9	1896	−48	18	22
			−40	10	26
			−46	18	32
L precuneus/inferior parietal lobule	7/40	1032	−28	−64	46
			−32	−48	44
R inferior frontal gyrus/insula	47/13	496	36	22	−12
			32	28	0
R inferior parietal lobule	40	336	38	−50	46
R inferior frontal gyrus	45	208	48	22	20
R insula	13	136	38	18	6
R insula	13	40	42	16	0
**CONTRAST: EMOTIONAL VERBAL > NON-EMOTIONAL VERBAL**					
No significant cluster					
**CONTRAST: NON-EMOTIONAL VERBAL > EMOTIONAL VERBAL**					
L inferior/middle frontal gyrus	9	680	−48	8	34
			−44	8	38
L precuneus	7	408	−8	−65	58
L precuneus	7	344	−7	−64	48
**CONJUNCTION: NON-EMOTIONAL VERBAL AND NON-EMOTIONAL SPATIAL**					
Pre-SMA/cingulate gyrus	6/32	1632	−4	10	50
			10	22	34
			−4	20	42
R inferior/middle frontal gyrus	45/46	856	48	16	24
L insula	13	472	−36	20	4
R superior parietal lobule	7	160	32	−54	46
R insula	13	112	30	22	4
**CONTRAST: NON-EMOTIONAL VERBAL > NON-EMOTIONAL SPATIAL**					
L inferior/middle frontal gyrus	44/46/9	7040	−45	11	31
			−44	26	18
L inferior parietal lobule/precuneus	40/7	3552	−33	−54	46
			−26	−66	40
			−24	−70	50
**CONTRAST: NON-EMOTIONAL SPATIAL > NON-EMOTIONAL VERBAL**					
R inferior parietal lobule	40	768	42	−34	57
R precuneus	7	360	23	−44	63
**CONJUNCTION: EMOTIONAL VERBAL AND NON-EMOTIONAL SPATIAL/CONJUCNTION: THE THREE DOMAINS**					
Cingulate gyrus	32	752	10	20	36
			2	20	42
L pre-SMA	6	328	−6	6	54
R inferior frontal gyrus	45	24	44	16	22
R superior parietal lobule	7	16	34	−54	46
R inferior frontal gyrus	47	8	50	18	−10
**CONTRAST: EMOTIONAL VERBAL > NON-EMOTIONAL SPATIAL**					
L inferior/middle frontal gyrus	9	1080	−43	7	29
			−40	10	26
			−45	16	32
Cingulate gyrus	32	440	12	22	46
L angular gyrus	39	336	−32	−54	45
**CONTRAST: NON-EMOTIONAL SPATIAL** > **EMOTIONAL VERBAL**					
R inferior parietal lobule	40	264	42	−36	62

L, left; R, right; BA, Brodmann's area.

**Figure 3:**
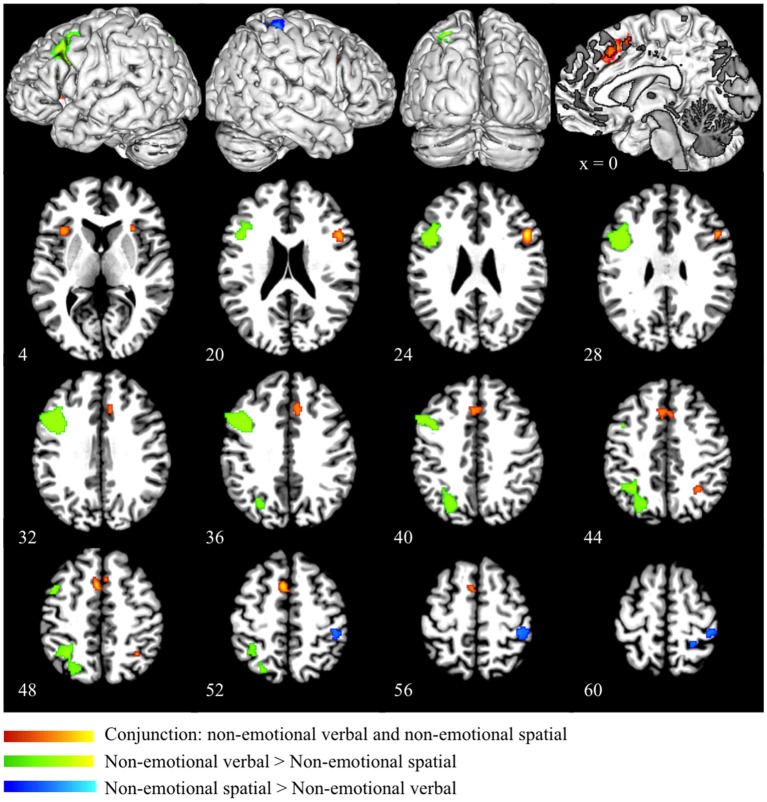
**Conjunction and subtraction analyses of non-emotional verbal and non-emotional spatial interference processing**. The scale bar in red represents minimum ALE values from 0 to 0.022 in the conjunction analysis. The scale bars in green represents *z*-values from 0 to 6 for the contrast of non-emotional verbal > non-emotional spatial and the scale bars in blue represents *z*-values from 0 to 6 for the contrast of non-emotional spatial > non-emotional verbal.

In the conjunction analysis of the non-emotional verbal and non-emotional spatial domain, we found common activation in the dorsal ACC (BA 32), pre-SMA (BA 6), bilateral insula (BA 13), right inferior frontal gyrus (BA 45), right DLPFC (BA 46) and right superior parietal lobule (BA 7) (as shown in red in Figure 3 and Table 3). Direct contrast between the two domains found higher convergence of activity for the non-emotional verbal domain in the left DLPFC (BA 46/9), left IFG (BA 44), and left PPC (BA 40/7) (as shown in green in Figure 3 and Table 3). In contrast, we found higher activation for non-emotional spatial interference in the right inferior parietal cortex (BA 40) and right precuneus (BA7) (as shown in blue in Figure 3 and Table 3).

**Figure 4:**
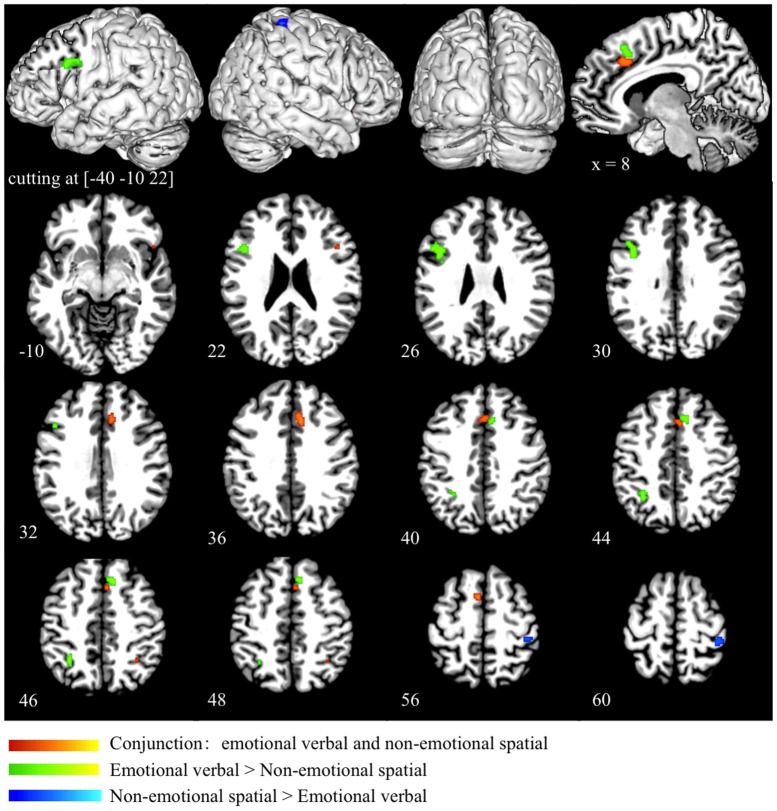
**Conjunction and subtraction analyses of emotional verbal and non-emotional spatial interference processing**. The scale bar in red represents minimum ALE values from 0 to 0.022 in the conjunction analysis. The scale bars in green represents *z*-values from 0 to 6 for the contrast of emotional verbal > non-emotional spatial and the scale bars in blue represents *z*-values from 0 to 6 for the contrast of emotional non-emotional spatial > emotional verbal.

Conjunction analysis of the emotional verbal and non-emotional spatial domain revealed significant convergence of activity mainly in the ACC (BA 32) and the pre-SMA (BA 6). Small clusters of overlapping activity were also found in the right IFG (BA 45/47) and right superior parietal lobule (BA 7) (as shown in red in Figure 4 and Table 3). Direct contrast between the two processing domains revealed higher convergence of activity for the emotional verbal processing in the left inferior and middle frontal gyrus (BA 9), right cingulate gyrus (BA 32) and left angular gyrus (BA 39) (as shown green in Figure 4 and Table 3). In contrast, greater activation was found for the non-emotional spatial interference in the right inferior parietal lobule at BA 40 (as shown in blue Figure 4 and Table 3).

Finally, we performed a conjunction analysis across all three domains and found that the overlap across the three domains was exactly the same as the overlap between emotional verbal and non-emotional spatial domain, as shown in red in Figure 4 and Table 3.

## Discussion

To understand how the nature and source of interference might influence neural activation, previous meta-analysis studies have identified neural systems associated with distinguishable stages or sub-components of interference processing (Nee et al., 2007; Niendam et al., 2012; Cieslik et al., 2015). Our study provided the first meta-analysis to examine whether the same neural networks are recruited across emotional and non-emotional interference processing. We conducted coordinate-based ALE meta-analyses and found that the dorsal ACC, pre-SMA, right anterior insular cortex and right IFG showed reliable convergence of activity across emotional and non-emotional interference processing. Moreover, direct comparisons across different domains found that frontal and parietal regions were preferentially associated with specific forms of interference processing.

Previous studies have reported that the dorsal ACC, labeled as middle cingulate cortex by some researchers (Vogt, 2005; Stevens et al., 2011), might be recruited in interference processing irrespective of verbal or spatial domains (Barch et al., 2001; Fan et al., 2003; Cieslik et al., 2015). The present study extended previous research and demonstrated converging activation in the dorsal ACC across emotional and non-emotional interference processing. The exact function of the ACC has been the subject of much debate (Barch et al., 2001; Mansouri et al., 2009), with one influential view postulating that the ACC monitors or detects the occurrence of conflict between task-relevant and task-irrelevant information and subsequently conveys the information to other region, such as DLPFC, to trigger control adjustments (Barch et al., 2001; Botvinick et al., 2001; Carter and Van Veen, 2007). According to this view, our results suggest that the dorsal ACC may be implicated in monitoring both emotional and non-emotional conflict and it signals other areas to implement cognitive control according to the requirements of specific tasks. An alternative view is that the ACC is part of the neurocircuitry that adjusts the level of cognitive control by biasing processing of the task relevant information (Posner and DiGirolamo, 1998; Roelofs et al., 2006). Evidence supporting this view is that besides an interference effect, ACC shows a facilitation effect, which is manifested as greater activation for neutral condition than congruent condition, both conditions not involving interference processing (Roelofs et al., 2006). We cannot examine this hypothesis in the current meta-analysis, because most of the studies selected did not reported brain activation associated with the contrast between neutral condition and congruent condition.

It has long been proposed that the ACC can be divided into the dorsal “cognitive” division, which is connected to the DLPFC, PCC, premotor and SMA, and the ventral “emotional” division, which is connected to amygdala, orbitofrontal, insula and so on (Vogt et al., 1992; Devinsky et al., 1995; Bush et al., 2000; Stevens et al., 2011). We did not find activation in the ventral ACC for emotional interference or larger activation in this region for the emotional relative to the non-emotional interference. One possible explanation is that that the facial expressions and emotional words in the incongruent condition are comparable to those in the congruent or neutral conditions in terms of emotional salience, and thus brain activity in the ventral ACC associated with emotional processing is no greater for the incongruent relative to the control conditions. An alternative explanation is that the ventral ACC may come to play a role in a specific stage of interference processing (e.g., conflict resolution), but its activity might be difficult to be detected by directly contrasting incongruent and control conditions. This account is supported by neuroimaging studies, in which trial-to-trial conflict resolution effects were analyzed (Etkin et al., 2006; Egner et al., 2008). It has been found that the ventral ACC made key contributions to the resolution of emotional interference, which was assessed by comparing incongruent trials that were preceded by an incongruent trial (high conflict resolution condition) with incongruent trials that were preceded by a congruent trial (low conflict resolution condition). Moreover, there is growing evidence showing that the dorsal ACC is involved in the appraisal and expression of negative emotion (Etkin et al., 2011), and experience of pain (Farrell et al., 2005), leading to the suggestion that cognitive control, negative affect, and pain may be integrated in the dorsal ACC (Shackman et al., 2011). Therefore, it is possible that the dorsal ACC might play an integral role in cognitive control and emotional processing during the face-word conflict task.

Our results also showed common activation in the right anterior insula across emotional and non-emotional interference processing, as revealed by the conjunction analyses of emotional verbal and non-emotional verbal domains, as well as non-emotional verbal and non-emotional spatial domains. Although we didn't find significant overlap in the anterior insula for emotional verbal and non-emotional spatial interference, there was a close correspondence between the two domains. The results were consistent with previous meta-analysis study by Cieslik et al. (2015), in which the right insula was commonly activated by the go/no-go task, stop signal task, Stroop and spatial interference task. The insula is implicated in both cognitive and affective functions (Craig, 2009; Menon and Uddin, 2010), and it is considered as an “integral hub,” linking information from diverse functional systems (Kurth et al., 2010; Medford and Critchley, 2010). Furthermore, the insula has been found to be structurally and functionally connected to the ACC (Dosenbach et al., 2007; Craig, 2009; Lerner et al., 2009; Taylor et al., 2009; Medford and Critchley, 2010; Menon and Uddin, 2010; Cauda et al., 2011). Dosenbach et al. suggest that the dorsal ACC and anterior insula form a core network that is crucial for the establishment and maintenance of task set (Dosenbach et al., 2006, 2007). Medford et al. further demonstrated information transfer between the two regions, postulating that the insula serves as an input and the ACC as an output component of a function system, in which the integrated awareness of cognitive, affective and physical state generated by the insula re-represented in the ACC as a basis for the selection and preparation for response (Medford and Critchley, 2010). Thus, the anterior insular cortex may serve to integrate information from different dimensions and then transfer information to the dorsal ACC to implement detection and monitoring functions during interference processing.

In the present study, the pre-SMA and the right IFG were also consistently activated across processing domains, indicating their domain-general role in interference processing. Previous research suggested that these two regions were crucial for response inhibition, where cognitive control is engaged to override prepotent responses and filter out irrelevant information (Bunge et al., 2002; Aron et al., 2003, 2014; Verbruggen and Logan, 2008; Sharp et al., 2010; Levy and Wagner, 2011). The pre-SMA, dorsal to the ACC, is located at the interface between prefrontal and motor systems and thus it is hypothesized to participate in higher functions related to executive control of motor, such as internally guided action (Lau et al., 2006), switching between action sets (Kennerley et al., 2004), sequential organization of movement (Tanji, 2001), resolution of competition between motor plans (Nachev et al., 2007; Mostofsky and Simmonds, 2008) and so on. Nachev et al. (2007) demonstrated that a patient with lesions to the right pre-SMA showed impairment in the ability to inhibit competing motor plans in situations of response conflict. Moreover, gray matter density in the pre-SMA was found to be positively correlated with the ability to voluntary select the correct action to resolve response conflict (van Gaal et al., 2011). Similarly, many neuroimaging studies have demonstrated that activation in the right IFG is associated with response inhibition and interference suppression (Bunge et al., 2002; Levy and Wagner, 2011; Aron et al., 2014). Patients with damage to this region were found to take longer to stop a prepotent response compared to healthy adults (Aron et al., 2003). However, the specific contributions of the pre-SMA and right IFG to response inhibition are controversial. Sharp et al. showed that the right IFG implements an attentional function whereas the pre-SMA implements an inhibitory control function (Sharp et al., 2010), but some other researchers proposed that the pre-SMA generates a control signal and right IFG implements the inhibition control (Aron, 2011). Accumulating evidence clearly indicates the critical role of the pre-SMA and right IFG in tasks that require response inhibition. Nonetheless, their precise functions during inhibitory processes are still unclear. In the incongruent condition of interference tasks, the activation of more than one potential response plan may lead to the recruitments of pre-SMA and right IFG to inhibit the inappropriate response and select the appropriate response.

We found activation in the DLPFC and PPC across interference domains, but with differing lateralization patterns and extent of activation. Brain activity in the DLPFC and PPC was predominantly left lateralized for the verbal interference, but right lateralized for spatial interference. The subtraction analyses also revealed higher convergence of activity in the left DLPFC and left PPC for the verbal than spatial interference, in contrast to higher convergence in the right PPC for the spatial than verbal interference. This phenomenon is probably due to left hemispheric specialization for processing verbal stimuli and right hemispheric specialization for spatial stimuli (Smith et al., 1996; Reuter-Lorenz et al., 2000; Thomason et al., 2009). The DLPFC is proposed to exert top-down control, leading to a bias in processing task-relevant information in the face of conflict (MacDonald et al., 2000; Egner and Hirsch, 2005; Mansouri et al., 2009). The role of PPC in interference resolution is controversial and it has been implicated in top-down regulation of attention (Roberts and Hall, 2008), action planning when there is conflict response (Coulthard et al., 2008) and cognitive control at the level of stimuli representation (Liston et al., 2006). Our results indicated that the DLPFC and PPC may exert cognitive control according to specific contexts and task requirements. In addition, the color-word Stroop task showed greater activation in the left DLPFC and left PPC than face-word task and spatial interference tasks. One explanation is that reading the color name may be a relatively automatic process, requiring greater demand for cognitive control to resolve the interference (MacLeod and MacDonald, 2000; Nee et al., 2007). Finally, it is noteworthy that the face-word conflict task activated bilateral fusiform gyri, which are recognized as crucial regions for face processing (Kanwisher et al., 1997). It reflects that interference resolution may involve amplification of the neural representations of task relevant information (Egner and Hirsch, 2005; Etkin et al., 2006; Bitan et al., 2007).

To conclude, we examined common and distinct neural systems for emotional and non-emotional interference processing by performing an ALE meta-analysis. In contrast to the traditional dichotomy of ACC functions, our results showed that the dorsal ACC was recruited for emotional and non-emotional interference. In addition, the right anterior insula, pre-SMA and right IFG were commonly activated by interference processing across emotional and non-emotional domains. The anterior insular cortex may serve to integrate information from different dimensions and work together with the dorsal ACC to detect and monitor conflict. The pre-SMA and right IFG may be recruited to inhibit the inappropriate and select the appropriate response. In contrast, the DLPFC and PPC showed different degrees of activation and distinct lateralization patterns for different processing domains, suggesting that these regions may implement cognitive control based on the specific task requirements.

## Author contributions

MX, GX, and YY designed research, MX analyzed data, and MX, GX, YY wrote the paper.

### Conflict of interest statement

The authors declare that the research was conducted in the absence of any commercial or financial relationships that could be construed as a potential conflict of interest.
